# Impact of Ultrasound on the Physicochemical and Structural Properties of Gluten Protein During Controlled Thermal Treatment

**DOI:** 10.3390/foods15203725

**Published:** 2026-10-19

**Authors:** Zhonghua Gu, Jinshu Dai, Zhanqian Ma, Hongli Yao, Jing Chen, Rong Zheng, Xianmeng Xu

**Affiliations:** School of Food and Biological Engineering, Bozhou University, Bozhou 236800, China

**Keywords:** ultrasound, gluten protein, thermal processing, physicochemical properties, structural characteristics

## Abstract

Gluten proteins are widely used in the food processing industry, but their low purity, poor water solubility, and limited thermal stability restrict broader applications. Ultrasound is a non-thermal physical technique that can modify protein functionality; however, its effects on gluten proteins during thermal processing remain insufficiently quantified. Therefore, in this study, ultrasound intensity (60, 90, and 120 W/L) combined with controlled thermal treatment (30, 55, and 80 °C) was systematically evaluated in a simplified gluten model system, providing direct evidence of ultrasound-induced alterations in intermolecular forces and structural properties. Ultrasound treatment facilitated the removal of starch and other non-protein components from the gluten aggregates, as evidenced by protein content increasing from 85.47% in the untreated control to 87.73% in the sample treated at 120 W/L and 30 °C, accompanied by a decrease in starch content from 3.52% to 1.84% (*p* < 0.05). The water absorption index increased significantly with increasing ultrasound intensity, reaching 2.95 g/g at 120 W/L and 30 °C compared with 2.80 g/g in the untreated control (*p* < 0.05); however, the solubility index decreased from 3.75% to 2.77% (*p* < 0.05). In addition, thermal weight loss increased and the degradation temperature decreased significantly after ultrasound treatment at 30 and 55 °C (*p* < 0.05), while no significant change was observed at 80 °C. Chemical interaction analysis also revealed that ultrasound disrupted hydrogen bonds and increased the free sulfhydryl content, suggesting a possible alteration of the disulfide bond balance. Collectively, these findings suggested that ultrasound modulated the functional and structural characteristics of gluten proteins by affecting noncovalent/covalent interactions and protein–matrix associations. Thus, ultrasound represents a promising and feasible approach for the targeted modification of gluten proteins in food processing applications.

## 1. Introduction

Gluten, a natural macromolecular protein complex derived from wheat flour, is recovered after the removal of starch, dietary fiber, and other water-soluble components. Gluten proteins contain a relatively high proportion of glutamine and proline but have limited contents of essential amino acids such as lysine, which restricts their nutritional quality compared with many animal-derived proteins [1]. Nevertheless, gluten proteins are globally abundant and low-cost plant proteins with unique viscoelastic properties that are difficult to replicate with other protein sources. Based on their differential solubility, they are classified into four fractions: albumins (soluble in water or dilute salt solutions), globulins (soluble in salt solutions but insoluble in water), gliadins (soluble in 70% ethanol), and glutenins (soluble in dilute acids or alkalis) [1,2]. Glutenin and gliadin, the two major fractions, are primarily responsible for dough viscoelasticity and extensibility. Glutenin is a heterogeneous macromolecular polymer composed of polypeptide chains linked by intermolecular disulfide bonds, whereas gliadin is a monomeric protein with a spherical conformation [3]. The demand for gluten-based products, including hydrolyzed peptides, texturized proteins, low-gluten bread, and frozen dough, has been steadily increasing. However, gluten proteins’ low purity, poor water solubility, limited thermal stability, and high content of hydrophobic amino acids limit their further applications. Through appropriate modifications, the functional properties of gluten proteins can be effectively enhanced, thereby broadening their applications in the food and feed industries.

Ultrasonic processing is generally classified as a non-thermal physical technique, although cavitation-induced energy dissipation can generate localized heating, and its application at elevated temperatures involves combined thermal and mechanical effects. Ultrasonic processing has been widely adopted in the food industry and has been shown to improve food product functionality through thermal, mechanical, and chemical (free radical) mechanisms [4,5,6,7,8]. Recent applications of ultrasound in gluten protein modification have demonstrated its potential to enhance processing quality [9,10]. Ultrasonication improves flour product quality through cavitation effects that promote gluten network formation, enhance dough rheological properties, and improve texture and flavor [11]. Beyond flour products, high-intensity ultrasound is widely applied in food processing operations such as cutting, freezing, drying, homogenization, and extraction [12], and in frozen wheat dough, multifrequency ultrasound disrupts starch amorphous regions via localized high-pressure cavitation, thereby increasing water retention and enhancing starch–water interactions [13]. Ultrasound treatment induced notable changes in hydrogen bonding, molecular crosslinking, granule size distribution, mechanical properties, and gas cell structures within protein matrices, ultimately leading to significant modifications in food quality [14,15,16]. Despite these promising findings, the specific influence of ultrasound intensity on gluten protein properties during thermal processing remains poorly understood.

Numerous studies have investigated the effects of ultrasound on gluten protein quality in complex systems, such as dough, frozen dough, and whole wheat kernels [11,12,13,14,15,16,17]. However, the influence of other exogenous components on gluten protein quality is difficult to exclude in such systems. Moreover, the specific influence of ultrasound intensity on gluten protein properties during thermal processing remains poorly understood. To overcome this limitation, a simplified model system composed predominantly of gluten proteins was developed to enable a direct assessment of ultrasound effects on gluten properties under controlled thermal processing conditions. The specific novelty of this study is the systematic combination of ultrasound intensity (60, 90, and 120 W/L) with controlled thermal treatment (30, 55, and 80 °C) in this simplified system, which allows the main effects of ultrasound intensity and temperature, as well as their interaction, to be evaluated without interference from other components. This approach differs from that taken in previous studies on complex dough or flour systems, in which the contribution of ultrasound alone could not be isolated. Accordingly, this study aimed to examine the influence of varying ultrasound intensities on gluten physicochemical properties and to elucidate ultrasound-induced alterations in intermolecular forces during thermal treatment.

## 2. Materials and Methods

### 2.1. Materials and Chemicals

Wheat gluten was kindly provided by Anhui Ante Food Co., Ltd. (Suzhou, Anhui, China). Its proximate composition was determined as follows: protein 80.33%, fat 0.56%, starch 7.72%, moisture 8.50%, and ash 0.94%. All other chemical reagents and solvents were of analytical grade unless otherwise specified.

### 2.2. Preparation of Ultrasound-Treated Wheat Gluten Proteins

Ultrasound-treated gluten proteins were prepared following the method of Zhang et al. [18] with minor modifications. The gluten suspension (150 g/L) was stirred with a magnetic stirrer, then preheated to and held at the desired temperature for 10 min. The suspension was then transferred to an ultrasonic reaction tank (GL1022, Jiangsu Jiangda Wukesong Biotechnology Co., Ltd., Zhenjiang, China) equipped with a circulating water bath to maintain the treatment temperature at 30, 55, or 80 °C. The suspension was treated at 40 kHz with varying power intensities (60, 90, and 120 W/L) for 30 min. The acoustic power delivered to the sample was calibrated by the calorimetric method, according to the procedure of Kimura et al. [19]. The energy densities corresponding to the nominal settings of 60, 90, and 120 W/L were 0.108, 0.162, and 0.216 kJ/mL, respectively, while the specific energies were 0.72, 1.08, and 1.44 kJ/g of gluten, respectively. The temperature was monitored throughout the treatment using a thermocouple to ensure that the set temperature was maintained, and the actual temperature variation was within ±1 °C. The treatment temperatures (30, 55, and 80 °C) were selected to represent the pre-, partial, and full denaturation stages of gluten proteins, respectively, and also correspond to the conditions used in dough mixing, fermentation, baking, and steaming, enabling a systematic evaluation of the combined effects of ultrasound and thermal processing. After treatment, the suspension was centrifuged at 3000× *g* for 10 min at room temperature. The supernatant containing released starch and soluble components was retained for mass balance determination, and the resulting precipitate was washed three times with deionized water to remove residual starch and water-soluble components. The washed precipitate was then freeze-dried, ground to pass through a 100-mesh sieve, sealed in airtight bags, and stored at 4 °C until further analysis. Ultrasonicated samples were designated GUS_60_, GUS_90_, and GUS_120_, with the subscripts indicating ultrasound intensities of 60, 90, and 120 W/L, respectively; water-bath-treated controls were labeled GW.

### 2.3. Protein and Starch Contents in Gluten Samples

The protein (N × 5.7) contents of gluten samples were determined using AACC International Method 46-13 (AACC, 2012) [20]. Total starch content was determined using a kit purchased from Megazyme International Ireland Ltd. (Wicklow, Ireland) following AACC Method 76-13 (AACC, 2012) [20].

### 2.4. Water Absorption Indices (WAI) and Solubility Indices (SI) in Gluten Samples

WAI and SI were determined according to the methods described by Delatte et al. and Ma et al. [21,22]. Gluten samples (2.0 g, dry basis) were transferred to 50 mL centrifuge tubes containing 10 mL of deionized water. The tubes were incubated in a water bath at 30 °C and continuously stirred for 30 min; then, they were centrifuged at 5000× *g* for 5 min. The supernatant was decanted and oven-dried at 105 °C until a constant weight was achieved, while the sediment was accurately weighed. WAI and SI were then calculated using the following equations:

WAI (g/g) = weight of the sediment/weight of dry basis

SI (%) = weight of supernatant dry basis/weight of dry basis × 100

### 2.5. Dynamic Rheological Measurement

Rheological properties of gluten samples were determined according to the method of Hu et al. [23] with minor modifications. Freeze-dried gluten samples were rehydrated with deionized water to a final concentration of 50% (*w*/*w*) and mixed thoroughly. The rehydrated samples were loaded onto a DHR rheometer (TA Discovery DHR-1, New Castle, DE, USA) with a 40 mm parallel plate and a 1 mm gap. Oscillatory mode was used to determine the linear viscoelastic region. Storage (G′) and loss modulus (G″) were measured at a strain of 1.0% within the linear viscoelastic region over a frequency range of 0.1–10 Hz.

### 2.6. Thermogravimetric Analysis (TGA)

TGA was performed on gluten protein samples using a thermal gravimetric analyzer (TA Instruments, New Castle, DE, USA) following the method of Khatkar et al. [24]. Approximately 10 mg of each sample was placed in a ceramic crucible and heated from 50 °C to 600 °C at 10 °C/min under nitrogen atmosphere. The degradation temperature and total weight loss were determined using the TGA instrument’s built-in software.

### 2.7. Free Sulfhydryl (SH) Content

Free sulfhydryl (SH) content was determined according to the methods of Yang et al. and Hong et al. [25,26] with minor modifications. Tris-gly buffer (pH 8.0) was prepared by dissolving Tris (20.836 g), glycine (13.512 g), and disodium EDTA (2.978 g) in deionized water (2000 mL). A Tris-gly-8 M urea solution was prepared by dissolving urea (480.480 g) in 1000 mL of the Tris-gly buffer. DTNB solution (4 mg/mL) was freshly prepared by dissolving 4 mg of DTNB in 1 mL of Tris-gly buffer.

For SH content determination, gluten samples (20 mg) were dissolved in 5 mL of Tris-gly-8 M urea solution, and 50 µL of DTNB solution was added. The mixture was incubated at 25 °C for 1 h, then centrifuged at 10,000× *g* for 10 min. The absorbance of the supernatant was measured at 412 nm. A sample-free Tris-gly buffer was used as the blank. Free SH content was calculated as follows:

Free SH μmol/g protein = (73.53 × A412 × D) ÷ C

where 73.53 = 106/(1.36 × 104), with 1.36 × 10^4^ M^−1^·cm^−1^ being the molar extinction coefficient of DTNB; A412, C, and D represent the sample absorbance, protein concentration (mg/mL), and dilution factor (5.02), respectively.

### 2.8. Noncovalent Bonds

Noncovalent bond contents in gluten samples were determined according to the method of Wang et al. [27] with minor modifications. Samples were treated with reagents that selectively disrupt hydrogen bonds and hydrophobic interactions. The solubility in different solutions was measured to evaluate the contributions of various chemical interactions. Selective buffers were prepared by adding different reagents to phosphate buffer (0.05 M, pH 7.0): PA (0.1 M NaCl), PB (0.6 M NaCl), PC (0.6 M NaCl + 1.5 M urea), and PD (0.6 M NaCl + 8 M urea).

Gluten samples equivalent to 1.00 mg of protein were weighed and mixed with 1.5 mL of each selective buffer (PA, PB, PC, and PD). The mixtures were vortexed at 25 °C for 1 h and then centrifuged at 10,000× *g* and 4 °C for 15 min. The protein concentration in the supernatant was determined using a BSA protein assay kit (Nanjing Jiancheng Biotechnology Co., Ltd., Nanjing, China). The contributions of different noncovalent interactions were calculated as follows:

Ionic bonds = protein solubility in PB—protein solubility in PA.

Hydrogen bonds = protein solubility in PC—protein solubility in PB.

Hydrophobic interactions = protein solubility in PD—protein solubility in PC.

All solubility values were expressed in mg/mL. This sequential subtraction approach allows the contribution of each interaction to be estimated based on the selective disrupting reagents in each buffer.

### 2.9. Fourier Transform Infrared (FTIR) Spectroscopy

FTIR spectroscopy was used to characterize the gluten samples’ secondary structures. The samples were mixed with KBr powder (1:100, *w*/*w*) and compressed into transparent pellets. Spectra were recorded on an FTIR spectrophotometer (Nicolet6700, Thermo Fisher Scientific, Waltham, MA, USA) over the range of 4000–400 cm^−1^ at a resolution of 4 cm^−1^ with 32 scans per spectrum. The secondary structure was analyzed based on the amide I region (1600–1700 cm^−1^). Data processing and peak fitting were carried out using Omnic 8.0 and Peakfit 4.12 software. Before peak fitting, all spectra were baseline-corrected using the automatic baseline correction function in Omnic 8.0 and normalized to the amide I band area. Peak fitting was performed in Peakfit 4.12 using Gaussian functions with fixed peak positions at 1612–1614, 1629–1632, 1640–1650, 1650–1660, 1665–1670, and 1687–1690 cm^−1^, allowing the peak width and amplitude to vary. The goodness of fit was evaluated by R^2^ values (﹥0.98 for all samples). The six bands were grouped into four categories: β-sheets (1612–1614 cm^−1^ and 1629–1632 cm^−1^), random coils (1640–1650 cm^−1^), α-helices (1650–1660 cm^−1^), and β-turns (1665–1670 cm^−1^ and 1687–1690 cm^−1^) [28].

### 2.10. Statistical Analysis

All experiments were performed in triplicate, with each replicate representing an independent ultrasound treatment on a separate batch of gluten suspension. Data were analyzed using SPSS 22.0 (SPSS Inc., Chicago, IL, USA), and significant differences between means were determined by two-way ANOVA followed by Duncan’s multiple range test at *p* < 0.05. Results are expressed as mean ± standard deviation.

## 3. Results and Analysis

### 3.1. Analysis of Chemical Composition

The protein and starch contents of the gluten samples shown in Table 1 refer to the final recovered gluten fraction after separation and washing, rather than the entire original suspension. The total recoveries of the gluten fraction (dry basis) ranged from 90.5% to 92.7% across all treatments (Table S1), indicating a good mass balance. The protein content increased significantly with rising treatment temperature and ultrasound intensity (Table 1). This was likely attributable to temperature-induced partial depolymerization of gluten proteins, which weakened protein–starch interactions and facilitated the removal of starch and other non-protein components from the gluten aggregates. Ultrasonication enlarged the pore size of the gluten matrix, reducing adhered substances such as starch and polysaccharides, with this effect strengthening at higher ultrasound intensities [29,30].

Conversely, the starch content decreased with increasing temperature at a given ultrasound intensity (Table 1), likely due to temperature-induced depolymerization of gluten structures and the consequent release of entrapped starch. Under the same temperature, higher ultrasound intensity likewise reduced starch content, which was attributed to cavitation-induced loosening of the gluten network and widening of its internal pores, thereby weakening starch-protein binding [31]. It is worth noting that the starch released into the supernatant and washing fractions was not quantified in this study; further investigations are needed to address this.

### 3.2. Water Absorption Indices and Solubility Indices of Gluten Samples

The water absorption index (WAI) and solubility index (SI) of the gluten samples are provided in Table 2. The WAI increased significantly with increasing treatment temperature (Table 2), which was probably attributable to the exposure of internal hydrophobic and polar groups. In addition, the partial gelatinization of residual starch and water-carbohydrate polar interactions may have further enhanced water absorption. These results are in agreement with those of previous reports suggesting that heat treatment significantly improves the water-holding capacity of gluten [22,29]. The WAI also increased at higher ultrasound intensities, likely due to cavitation-induced depolymerization of gluten. This enlarged internal pores and exposed polar side chains, facilitating water penetration and its entrapment within the protein network [32].

By contrast, SI was significantly reduced by ultrasound treatment compared with the control (GW, Table 2), probably due to the release of starch granules entrapped within the gluten matrix and the dissociation of polysaccharides. Interestingly, SI exhibited a non-monotonic response to temperature, initially increasing and then declining at higher temperatures (Table 2). This biphasic behavior may be explained by heat-induced denaturation of gluten proteins, which formed a surface barrier around starch and other components, thereby restricting the release of soluble substances into the aqueous phase and ultimately reducing solubility [21].

### 3.3. Dynamic Rheological Analysis

**Figure 1:**
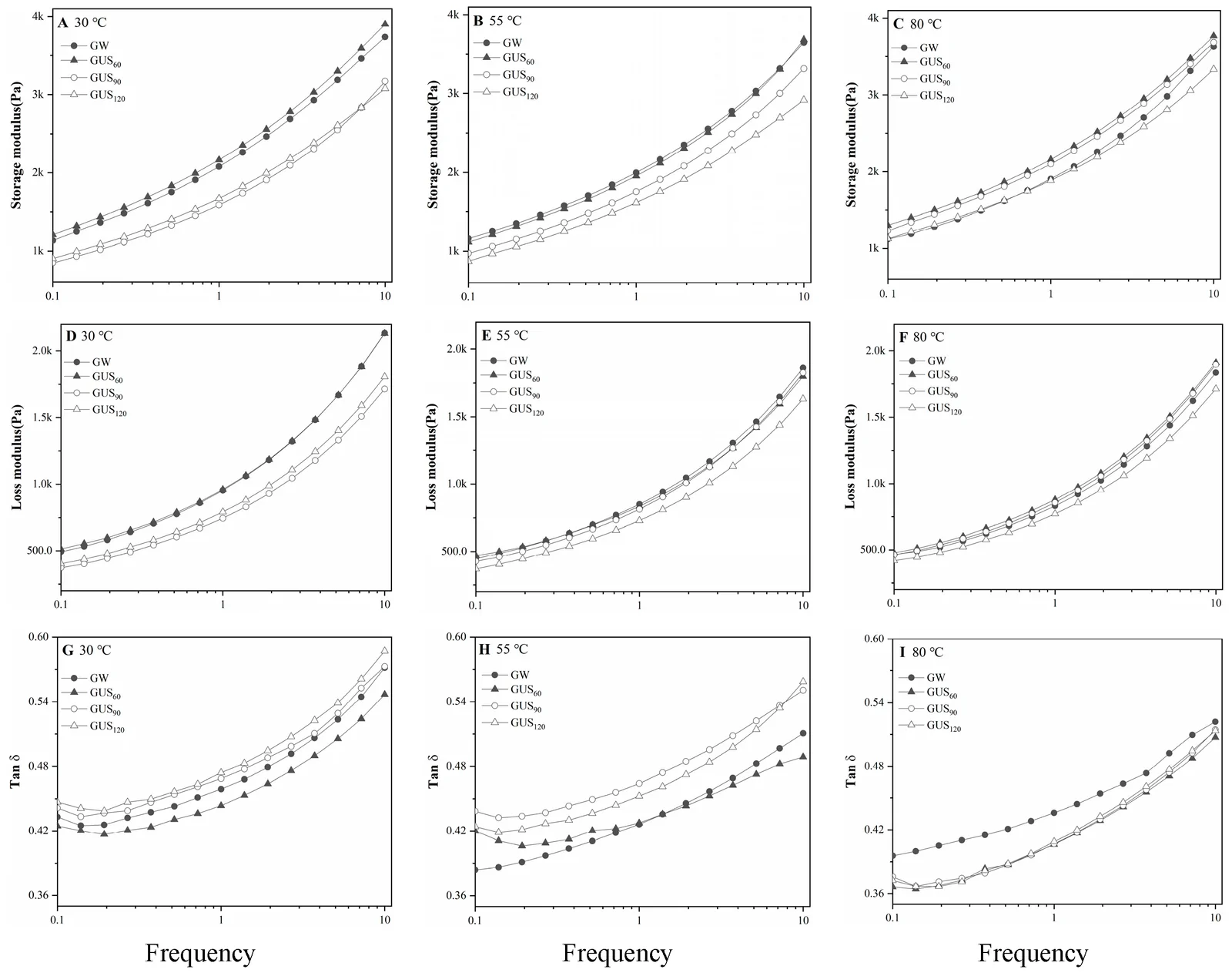
Frequency sweeps of storage modulus (G′, (**A**–**C**), loss modulus (G′′, (**D**–**F**), and loss tangent (tan δ, (**G**–**I**) of gluten samples treated at different ultrasonic intensities at various temperatures.

The frequency-dependent rheological behavior of gluten samples treated with varying ultrasound intensities (60 W/L, 90 W/L, and 120 W/L) at 30 °C, 55 °C, and 80 °C is shown in Figure 1. Under all tested conditions, the storage modulus (G′) exceeded the loss modulus (G″) (G′ > G″, Figure 1), indicating that all of the treated gluten samples exhibited a well-developed solid-like behavior. As shown in Figure 1A–F, G′ and G″ decreased slightly when the temperature increased from 30 °C to 55 °C, which was associated with the disruption of hydrogen bonds and the rearrangement of hydrophobic interactions at elevated temperatures. As the temperature rose from 55 °C to 80 °C, exposed SH groups underwent oxidation to form new disulfide bonds, facilitating intra- and intermolecular crosslinking of gluten proteins and thereby stabilizing the overall protein conformation. Consequently, no pronounced upward or downward shift in viscoelasticity was observed, consistent with the findings of Si et al. [33]. In addition, the G′ and G″ of the gluten samples decreased with increasing ultrasound intensity compared with the control, particularly at 90 W/L and 120 W/L, which was attributable to ultrasound cavitation. This result is consistent with previous reports that high-intensity ultrasound treatment loosens the gluten structure and enlarges its internal voids, leading to decreased viscoelasticity [32].

The loss tangent (tan δ = G″/G′) serves as a sensitive indicator of gluten viscoelasticity and is widely used to assess gluten protein quality, with higher values reflecting greater structural deterioration [34]. As illustrated in Figure 1G–I, tan δ declined with increasing temperature, which was probably attributable to the distinct thermal denaturation behaviors of glutenin and gliadin. At 30 °C and 55 °C, tan δ exhibited a biphasic response to increasing ultrasound intensity, decreasing initially and then increasing (Figure 1G,H). This result is consistent with the findings of Chemat et al. [35], who reported that low-intensity ultrasound induces a transient reduction in viscosity, whereas high-intensity ultrasound causes permanent depolymerization and irreversible rheological alterations. Collectively, these results indicate that moderate ultrasound intensity can lower tan δ, suggesting that optimized ultrasound treatment may alter the balance between the elastic and viscous behavior of gluten proteins. However, the decrease in both G′ and G″ indicates that ultrasound treatment also reduced the overall viscoelastic moduli, and therefore, the rheological results should not be interpreted as a simple improvement in gluten processing quality. The practical implications of these changes require further validation in real dough systems.

### 3.4. Thermal Analysis

The thermal properties of the gluten samples are summarized in Table 3. The occurrence of weight loss at 600 °C primarily reflects the thermal decomposition behavior and composition of the samples, and may also be influenced by the openness of the gluten network [24]. In this study, therefore, the weight loss percentage and degradation temperature (Td) extracted from the TGA profiles were used together as complementary indicators to compare the thermal decomposition behavior of gluten samples treated under different ultrasound intensities and temperatures. As shown in Table 3, weight loss increased significantly with ultrasound intensity at 30 °C and 55 °C, but remained unchanged at 80 °C, suggesting that ultrasound promoted weight loss when the treatment temperature was below the thermal denaturation temperature of gluten.

A significant decrease in the degradation temperature (Td) was observed for ultrasound-treated gluten samples at 30 °C and 55 °C, but no obvious change was detected at 80 °C (Table 3). This was likely attributable to ultrasound-induced alterations in intermolecular interactions, which render the gluten network less solid and stable when proteins are not yet denatured. These results suggest that ultrasound treatment reduced the thermal stability of gluten proteins below the thermal denaturation temperature, with the effect becoming more pronounced at higher ultrasound intensities. However, these interpretations are based on weight loss and Td data, and DTG curves would provide more detailed information on degradation kinetics. These findings are consistent with previous reports that high-intensity ultrasound alters the conformation of plant-based proteins and reduces their thermal stability by disrupting hydrogen bonds and hydrophobic interactions [27,35].

### 3.5. Analysis of Chemical Interactions

**Figure 2:**
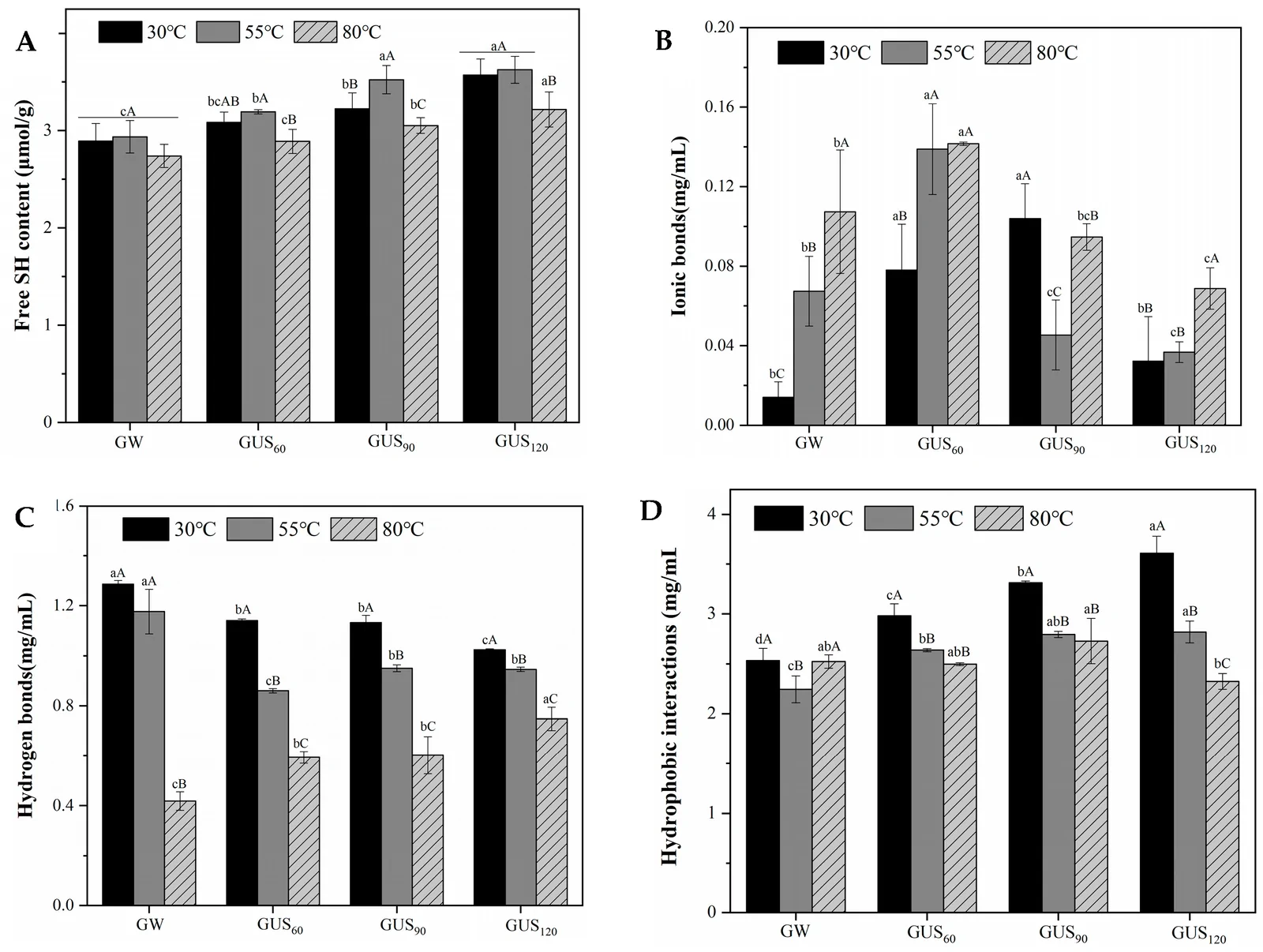
Effect of ultrasound treatment on the chemical interactions of gluten samples at various temperatures (**A**–**D**). Note: Different lowercase letters within the same bar color indicate significant differences with different ultrasonic intensities, and different capital letters indicate significant differences at various temperatures (*p* < 0.05). Data are presented as mean ± standard deviation (*n* = 3). Error bars represent standard deviation.

The free sulfhydryl (SH) content is widely used as a key indicator of gluten aggregation behavior and disulfide bond dynamics [36]. As shown in Figure 2A, the SH content increased slightly when the temperature was raised from 30 °C to 55 °C, reflecting the slow release of SH groups during gluten depolymerization. This trend is consistent with previous studies reporting an increase in free SH content in gluten heated at 25–60 °C [33,37]. The SH content decreased significantly when the temperature was raised further from 55 °C to 80 °C. This finding is consistent with that of Hwang et al. [38], who reported that temperature-induced unfolding of gluten proteins exposes SH groups that are subsequently oxidized to form inter- or intramolecular disulfide bonds. The ultrasound treatment significantly increased the free SH content compared with the control, likely due to the exposure of buried SH groups. At 30 °C, the free SH content increased by 7.6%, 13.4%, and 20.2% for GUS_60_, GUS_90_, and GUS_120_, respectively, compared with the control. At 55 °C, the corresponding increases were 6.9%, 15.8%, and 17.7% (Figure 2A). Notably, higher ultrasound intensities led to greater SH release, which was consistent with the findings of Zhang et al. [32], who attributed this effect to intense cavitation during triple-frequency ultrasound treatment. Collectively, these results indicate that both thermal and ultrasonic treatments modulate the SH/disulfide balance in gluten proteins, with ultrasound promoting SH exposure and high temperature favoring disulfide bond formation. It should be noted, however, that the free SH content alone does not directly quantify disulfide bonds, and the possibility that ultrasound inhibits disulfide bond formation is inferred rather than directly demonstrated. More studies are needed to clarify the changes in disulfide bonds that occur during ultrasound treatment.

The noncovalent bonds in gluten proteins primarily include ionic bonds, hydrophobic interactions, and hydrogen bonds. The noncovalent bonds in the treated gluten samples were assessed by measuring gluten solubility in selective solutions [39]. Gluten proteins exhibited low solubility in PA and PB solutions following ultrasonic and thermal treatment (Figure 2B), indicating that the treated samples contained few ionizable amino acid residues and exhibited relatively weak ionization [39]. As shown in Figure 2B, ionic bond strength initially increased and then decreased with increasing ultrasound intensity at the same treatment temperature. This result suggests that appropriate ultrasound intensity can enhance ionic bond strength, thereby maintaining the conformational stability of gluten proteins.

**Figure 3:**
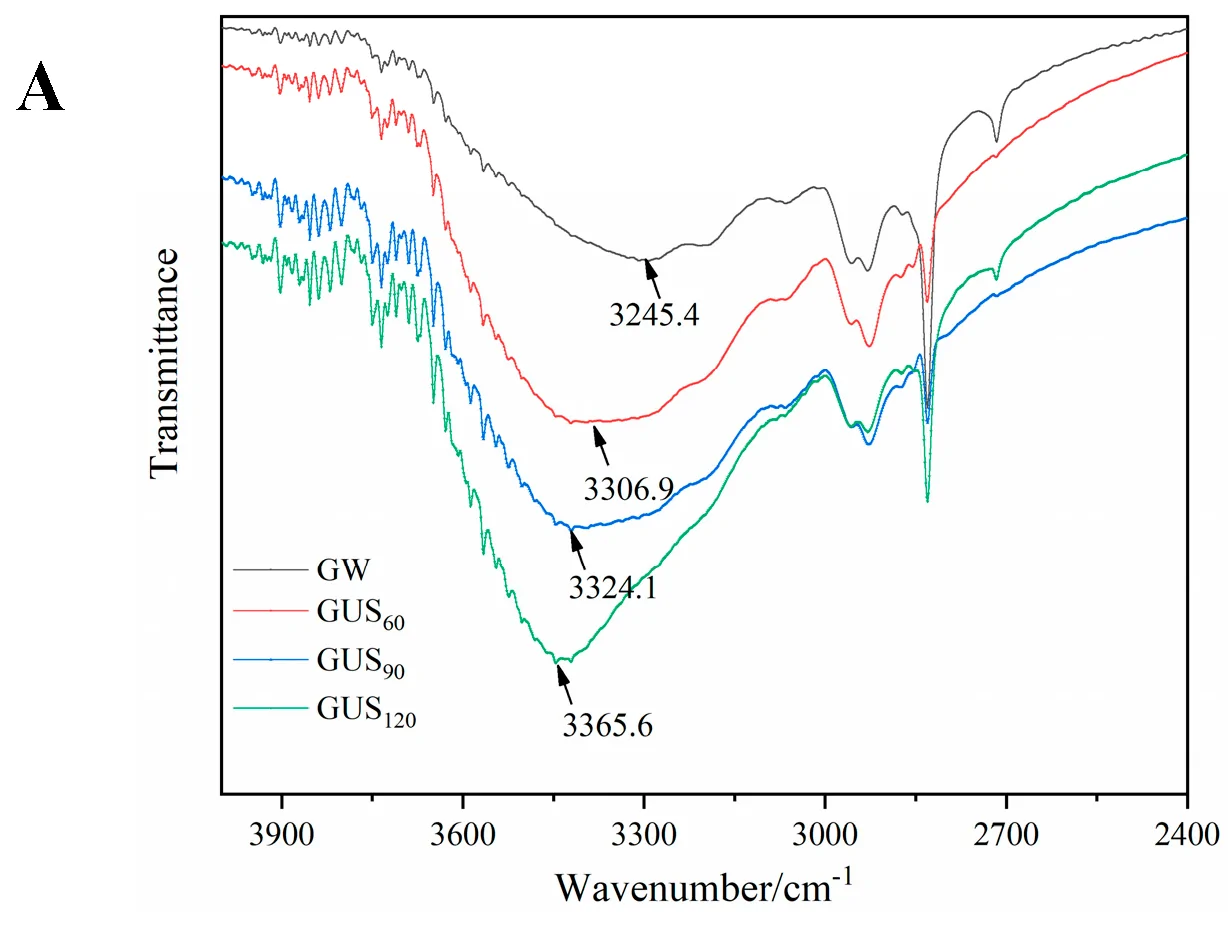

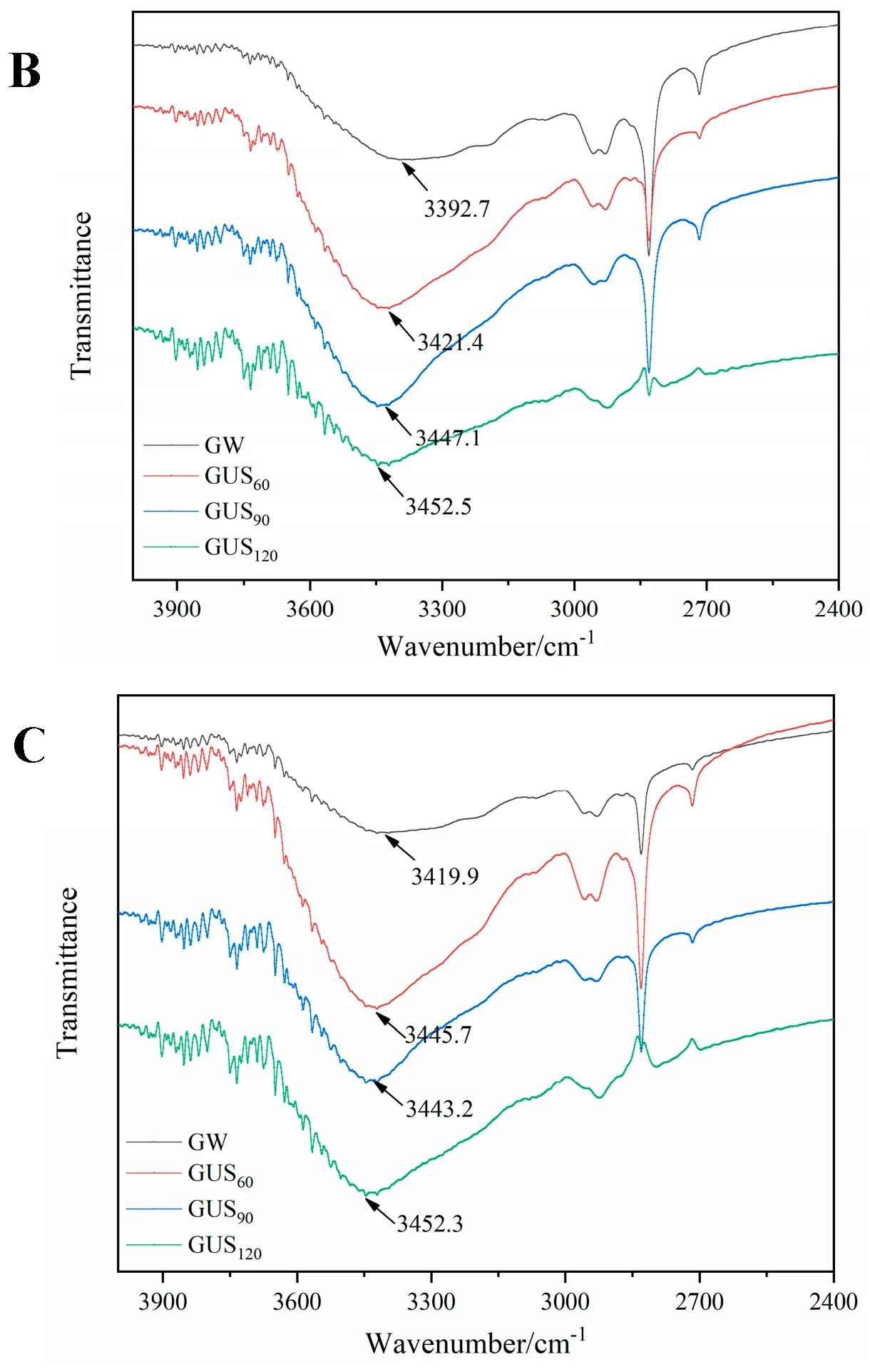
FTIR spectra of gluten samples treated with ultrasound at 30 °C (**A**), 55 °C (**B**), and 80 °C (**C**) for 30 min.

Solubility in PB and PC reflects hydrogen bond distribution in treated gluten samples, as urea disrupts hydrogen bonds and promotes protein solvation. As shown in Figure 2C, hydrogen bonds weakened with increasing treatment temperature. This result is consistent with that of Wang et al. [27], who reported that hydrogen bond contributions decreased from 4.133 g/L to 2.733 g/L as temperature increased from 25 °C to 90 °C. In addition, hydrogen bond strength decreased with increasing ultrasound intensity, with reductions of 15.2%, 17.4%, and 23.5% for GUS_60_, GUS_90_, and GUS_120_, respectively, at 30 °C compared with the control (Figure 2C). This might be attributed to ultrasound’s polarization effect [35]. Hydrogen bonds were further analyzed using FTIR spectroscopy. The absorption band corresponding to hydroxyl groups (-OH), associated with intramolecular hydrogen bonds, appeared between 3200 cm^−1^ and 3500 cm^−1^. A redshift indicates an increase in intermolecular hydrogen bonds, whereas a blueshift indicates a decrease [18]. As shown in Figure 3, the hydroxyl group band exhibited a blueshift after ultrasound treatment at 30 °C, with peak positions shifting from 3245.4 cm^−1^ (GW) to 3306.9, 3324.1, and 3365.6 cm^−1^ for GUS_60_, GUS_90_, and GUS_120_, respectively, indicating weakening of intramolecular hydrogen bonds. At 55 °C, the corresponding peak positions were 3329.7 cm^−1^ (GW), 3421.4 cm^−1^ (GUS_60_), 3447.1 cm^−1^ (GUS_90_), and 3452.5 cm^−1^ (GUS_120_), which did not show a monotonic trend with increasing ultrasound intensity. At 80 °C, the peak positions were similar among all samples, consistent with the fact that gluten proteins are extensively denatured at this temperature. Thus, the ultrasound-induced blueshift was most evident at 30 °C, whereas at higher temperatures its effect on hydrogen bonding was less pronounced and non-monotonic. These FTIR results were consistent with the solubility data shown in Figure 2C.

The following discussion of hydrophobic interactions covers two aspects: temperature effects at fixed ultrasound intensity and intensity effects at fixed temperature. As shown in Figure 2D, high-temperature treatment did not significantly alter hydrophobic interactions in the control group (GW); however, a significant reduction was observed in ultrasound-treated samples, indicating that ultrasound promoted the exposure of hydrophobic groups at lower temperatures. Furthermore, the hydrophobic interactions intensified with rising ultrasound intensity at a fixed temperature, with increases of 17.2%, 25.2%, and 33.6% for GUS_60_, GUS_90_, and GUS_120_, respectively, at 30 °C compared with the control (Figure 2D), likely due to turbulence, shear forces, and micro-fluidization from cavitation, which expose hydrophobic residues within the gluten matrix [40]. These findings are in agreement with those of Zhang et al. [32], who demonstrated that multi-frequency sonication significantly increased the surface hydrophobicity of wheat gluten proteins.

### 3.6. Secondary Structure of Gluten Proteins

FTIR spectroscopy provides information on the chemical microenvironment of protein side chains and polypeptide backbones. Specifically, analyzing the amide I band (1600–1700 cm^−1^) is widely used to monitor changes in secondary structures (α-helices, β-sheets, β-turns, and random coils) [40,41].

The secondary structure contents of gluten proteins are summarized in Table 4, and the original FTIR spectra together with the deconvoluted component peaks of the samples are presented in Figure S1. As shown in Table 4, the α-helix content decreased significantly with increasing temperature, whereas the β-sheet content increased. This is consistent with the fact that α-helices are predominantly stabilized by hydrogen bonds, whereas β-sheets are governed by both hydrophobic interactions and hydrogen bonding [42]. These findings are in line with the trends presented in Figure 2C,D. Additionally, ultrasound treatment decreased the α-helix content while increasing the β-sheet content, and these structural changes became more pronounced with increasing ultrasound intensity (Table 4). A moderate increase in random coil content was also noted in the ultrasound-treated samples, suggesting a more open protein structure conformation [43]. These findings align with those of Zhang et al. [32], who reported decreased α-helix content and increased β-sheet and β-turn content upon ultrasonication.

β-sheet structures are characteristic of glutenin, whereas α-helices are representative of gliadin [28]. Collectively, these data indicate that ultrasound disrupts ordered secondary structures and favors disordered conformations. The unfolding of polypeptide chains exposes both hydrophilic and hydrophobic residues to a new microenvironment, and the resulting structural reorganization is likely modulated by newly formed molecular crosslinks within and between gliadin and glutenin molecules, ultimately leading to altered gluten protein architecture.

## 4. Conclusions

This study provides new insight into the combined effects of ultrasound intensity and thermal treatment on gluten proteins in a simplified model system, in which the influence of other components was excluded. Ultrasound facilitated the removal of starch and other non-protein components from the gluten aggregates, thereby improving gluten purity and leading to increased water absorption and reduced solubility. Thermal analyses demonstrated that ultrasound treatment increased weight loss and decreased the degradation temperature of gluten proteins. The rheological analysis showed that ultrasound treatment decreased both G′ and G″ and altered tan δ in a temperature-dependent manner, indicating a modification of the viscoelastic behavior of gluten proteins rather than a simple improvement in processing quality. Analyzing the chemical interactions and FTIR spectroscopy revealed that ultrasound treatment increased ionic bonding and hydrophobic interactions, while reducing the content of α-helices, and increasing that of β-sheets. The ultrasound-induced weakening of intramolecular hydrogen bonds was most evident at 30 °C, whereas at higher treatment temperatures the effect was less pronounced. These changes were closely associated with the treatment temperature during ultrasound processing. Overall, ultrasound treatment represents a potential technique for modifying wheat gluten proteins in food processing applications. However, further studies are needed to validate these findings in complex food matrices and to evaluate the industrial feasibility of scale-up.

## Figures and Tables

**Table 1:** Protein and starch contents of gluten samples treated with different ultrasonic intensities at various temperatures ^1,2,3^.

Samples	Protein Content (%)			Starch Content (%)		
	30 °C	55 °C	80 °C	30 °C	55 °C	80 °C
GW	85.47 ± 0.55 ^cB^	86.21 ± 0.57 ^cB^	88.22 ± 0.36 ^aA^	3.52 ± 0.01 ^aB^	2.63 ± 0.09 ^aA^	2.44 ± 0.10 ^aB^
GUS_60_	86.33 ± 0.10 ^bcB^	87.26 ± 0.01 ^bAB^	89.37 ± 1.42 ^aA^	2.14 ± 0.06 ^bC^	2.00 ± 0.03 ^bB^	1.72 ± 0.07 ^bA^
GUS_90_	86.98 ± 0.34 ^bcB^	87.48 ± 0.09 ^bB^	88.69 ± 0.81 ^aA^	1.81 ± 0.06 ^cA^	1.87 ± 0.03 ^cB^	1.62 ± 0.02 ^bA^
GUS_120_	87.73 ± 0.36 ^aA^	88.53 ± 0.29 ^aA^	88.42 ± 1.01 ^aA^	1.84 ± 0.13 ^cB^	1.64 ± 0.02 ^cA^	1.58 ± 0.01 ^bA^

Note: ^1^ GW, GUS_60_, GUS_90_, and GUS_120_ represent gluten samples subjected to ultrasound at 0, 60, 90, and 120 W/L, respectively, in a water bath at 30 °C, 55 °C, and 80 °C for 30 min. ^2^ Values are presented as mean ± standard deviation (*n* = 3). Different lowercase letters in the same column indicate significant differences with different ultrasonic intensities, and different capital letters in the same row indicate significant differences at various temperatures (*p* < 0.05). ^3^ Two-way ANOVA: temperature effect (T), *p* < 0.01; ultrasound intensity effect (U), *p* < 0.01; T × U interaction, *p* < 0.05 for both protein and starch contents. Detailed results are provided in Supplementary Table S2.

**Table 2:** Water absorption (WAI) and solubility indices (SI) of gluten samples ^1,2,3^.

Samples	WAI (g/g)			SI (%)		
	30 °C	55 °C	80 °C	30 °C	55 °C	80 °C
GW	2.80 ± 0.01 ^bB^	2.89 ± 0.04 ^bAB^	3.08 ± 0.03 ^bA^	3.75 ± 0.11 ^aB^	3.93 ± 0.02 ^aA^	3.64 ± 0.08 ^aB^
GUS_60_	2.85 ± 0.06 ^bC^	2.94 ± 0.01 ^aB^	3.14 ± 0.01 ^bA^	3.35 ± 0.02 ^aC^	3.63 ± 0.11 ^baA^	3.50 ± 0.06 ^abB^
GUS_90_	2.93 ± 0.03 ^aB^	2.94 ± 0.00 ^aB^	3.21 ± 0.04 ^abA^	3.27 ± 0.11 ^aB^	3.33 ± 0.01 ^bA^	3.32 ± 0.13 ^bAB^
GUS_120_	2.95 ± 0.01 ^aB^	2.96 ± 0.00 ^aB^	3.23 ± 0.01 ^aA^	2.77 ± 0.15 ^bB^	2.99 ± 0.13 ^cA^	3.01 ± 0.16 ^cA^

Note: ^1^ GW, GUS_60_, GUS_90_, and GUS_120_ represent gluten samples subjected to ultrasound at 0, 60, 90, and 120 W/L, respectively, in a water bath at 30 °C, 55 °C, and 80 °C for 30 min. ^2^ Values are presented as mean ± standard deviation (*n* = 3). Different lowercase letters in the same column indicate significant differences with different ultrasonic intensities, and different capital letters in the same row indicate significant differences at various temperatures (*p* < 0.05). ^3^ Two-way ANOVA: temperature effect (T), *p* < 0.01; ultrasound intensity effect (U), *p* < 0.01; T × U interaction, *p* < 0.05 for both WAI and SI. Detailed results are provided in Supplementary Table S2.

**Table 3:** Weight loss at 600 °C and degradation temperature (Td) of gluten proteins analyzed from TGA profiles ^1,2,3^.

Samples	Weight Loss (%)			Td (°C)		
	30 °C	55 °C	80 °C	30 °C	55 °C	80 °C
GW	76.5 ± 0.3 ^b^	77.0 ± 0.3 ^c^	78.3 ± 0.4 ^b^	323.6 ± 1.4 ^a^	316.3 ± 1.1 ^a^	320.4 ± 0.6 ^a^
GUS_60_	77.0 ± 0.2 ^ab^	78.7 ± 0.4 ^b^	78.4 ± 0.5 ^b^	315.9 ± 0.9 ^c^	316.3 ± 1.3 ^a^	320.7 ± 0.8 ^a^
GUS_90_	78.4 ± 0.9 ^a^	78.0 ± 1.3 ^b^	78.9 ± 1.1 ^ab^	317.8 ± 1.1 ^b^	312.1 ± 0.8 ^b^	319.7 ± 2.4 ^a^
GUS_120_	78.7 ± 0.6 ^a^	79.8 ± 0.4 ^a^	79.4 ± 0.6 ^a^	315.1 ± 0.6 ^c^	309.9 ± 0.6 ^c^	320.9 ± 1.2 ^a^

Note: ^1^ GW, GUS_60_, GUS_90_, and GUS_120_ represent gluten samples subjected to ultrasound at 0, 60, 90, and 120 W/L, respectively, in a water bath at 30 °C, 55 °C, and 80 °C for 30 min. ^2^ Values are presented as mean ± standard deviation (*n* = 3). Different lowercase letters in the same column indicate significant differences with different ultrasonic intensities (*p* < 0.05). ^3^ Two-way ANOVA: temperature effect (T), *p* < 0.01; ultrasound intensity effect (U), *p* < 0.01; T × U interaction, *p* < 0.05 for both weight loss and Td. Detailed results are provided in Supplementary Table S2.

**Table 4:** Secondary structural compositions of gluten proteins treated with different ultrasonic intensities at various temperatures ^1,2,3^.

Samples	Secondary Structures (%)			
	α-Helix	β-Sheet	β-Turn	Random Coil
	30 °C			
GW	26.46 ± 1.04 ^aB^	31.50 ± 1.28 ^bA^	26.07 ± 0.76 ^aB^	15.95 ± 0.94 ^cC^
GUS_60_	25.01 ± 0.32 ^bB^	32.46 ± 0.98 ^abA^	26.15 ± 0.31 ^aB^	16.39 ± 0.97 ^bC^
GUS_90_	24.58 ± 0.26 ^bB^	33.23 ± 0.68 ^aA^	25.28 ± 0.86 ^abB^	16.90 ± 1.06 ^bC^
GUS_120_	24.20 ± 1.66 ^bB^	33.32 ± 0.01 ^aA^	24.24 ± 2.50 ^bB^	18.23 ± 1.13 ^aC^
	55 °C			
GW	23.54 ± 0.81 ^aB^	33.70 ± 0.26 ^aA^	24.40 ± 1.99 ^aB^	16.85 ± 1.30 ^cC^
GUS_60_	22.96 ± 0.79 ^aC^	33.83 ± 0.28 ^aA^	25.38 ± 1.38 ^aB^	19.33 ± 1.99 ^aD^
GUS_90_	22.63 ± 1.01 ^abC^	34.41 ± 0.73 ^aA^	24.88 ± 1.43 ^aB^	18.93 ± 1.43 ^bD^
GUS_120_	21.84 ± 0.88 ^bC^	34.67 ± 1.54 ^aA^	24.53 ± 2.3 ^aB^	19.47 ± 1.34 ^aD^
	80 °C			
GW	23.44 ± 0.03 ^aB^	35.44 ± 1.81 ^aA^	23.25 ± 1.10 ^aB^	18.86 ± 1.17 ^aC^
GUS_60_	22.83 ± 0.77 ^aB^	36.43 ± 1.11 ^aA^	22.83 ± 1.14 ^aB^	18.90 ± 1.09 ^aC^
GUS_90_	22.40 ± 0.35 ^aB^	36.69 ± 1.86 ^aA^	22.52 ± 1.14 ^aB^	19.52 ± 1.12 ^aC^
GUS_120_	22.54 ± 0.45 ^aB^	36.92 ± 2.67 ^aA^	22.11 ± 1.97 ^aB^	19.44 ± 1.75 ^aC^

Note: ^1^ GW, GUS_60_, GUS_90_, and GUS_120_ represent gluten samples subjected to ultrasound at 0, 60, 90, and 120 W/L, respectively, in a water bath at 30 °C, 55 °C, and 80 °C for 30 min. ^2^ Values are presented as mean ± standard deviation (*n* = 3). Different lowercase letters in the same column indicate significant differences with various ultrasonic intensities at the same temperature, and different capital letters in the same row indicate significant differences among secondary structures of the same sample (*p* < 0.05). ^3^ Two-way ANOVA: temperature effect (T), *p* < 0.01; ultrasound intensity effect (U), *p* < 0.01; T × U interaction, *p* < 0.05 for α-helix and β-sheet contents. Detailed results are provided in Supplementary Table S2.

## Data Availability

The data presented in this study are available on request from the corresponding author. The data are not publicly available due to institutional data policy.

## Supplementary Materials

The following supporting information can be downloaded at: https://www.mdpi.com/article/10.3390/foods15203725/s1, Table S1: Soluble solids and Recovery of gluten samples treated with different ultrasonic intensities at various temperatures; Table S2: Two-way ANOVA results (*p* values) for the main effects of temperature and ultrasound intensity and their interaction on the main responses; Figure S1: FTIR spectra in the amide I region (1600–1700 cm^−1^) and deconvoluted component peaks of gluten samples. (A–C) FTIR spectra at 30, 55, and 80 °C; (a–c) deconvoluted FTIR spectra at 30, 55, and 80 °C.
