# Supplementary material for: Impact of Ultrasound on the Physicochemical and Structural Properties of Gluten Protein During Controlled Thermal Treatment
Source: Foods. 2026 Oct 19;15(20):3725. doi: 10.3390/foods15203725 (PMC13704420; doi:10.3390/foods15203725)
Supplement: Supplementary file 1 [file foods-15-03725-s001.zip › foods-4583198-supplementary.pdf]

Table S1. Soluble solids and Recovery of gluten samples treated with different ultrasonic intensities at various temperatures.[1,2]

Sample	Soluble solids (%)	Recovery (%)
	30°C	
GW	7.3[b]	92.7
GUS_{60}	7.4[b]	92.0
GUS_{90}	7.9[a]	92.3
GUS_{120}	8.1[a]	91.5
	55°C	
GW	7.7[b]	92.1
GUS_{60}	7.9[b]	91.5
GUS_{90}	8.3[a]	91.5
GUS_{120}	8.5[a]	90.8
	80°C	
GW	8.3[c]	91.3
GUS_{60}	8.8[b]	90.8
GUS_{90}	9.4[a]	90.8
GUS_{120}	9.4[a]	90.5

Note: [1] GW, GUS_{60}, GUS_{90}, and GUS_{120} represent gluten samples subjected to ultrasound at 0, 60, 90, and 120 W/L, respectively, in a water bath at 30 °C, 55 °C, and 80 °C for 30 min.
[2] Values are presented as mean ± standard deviation. Different lowercase letters in the same column indicate significant differences with various ultrasonic intensities at the same temperature ($p < 0.05$).

Table S2. Two-way ANOVA results (*p* values) for the main effects of temperature and ultrasound intensity and their interaction on the main responses.[1,2]

Response	Temperature (T)	Ultrasound intensity (U)	T×U
Protein content	<0.01	<0.01	0.012
Starch content	<0.01	<0.01	0.018
WAI	<0.01	<0.01	0.022
SI	<0.01	<0.01	0.015
Weight loss	<0.01	<0.01	0.033
Td	<0.01	<0.01	0.026
Free SH content	<0.01	<0.01	0.014
Ionic bonds	<0.01	<0.01	0.042
Hydrogen bonds	<0.01	<0.01	0.025
Hydrophobic interactions	<0.01	<0.01	0.017
α-helix	<0.01	<0.01	0.039
β-sheet	<0.01	<0.01	0.031

Note: WAI, Water absorption index; SI, Solubility index; Td, Degradation temperature; Values are *p* values obtained from two-way ANOVA (n = 3). Temperature and ultrasound intensity were treated as fixed factors, and their interaction term was included in the model.

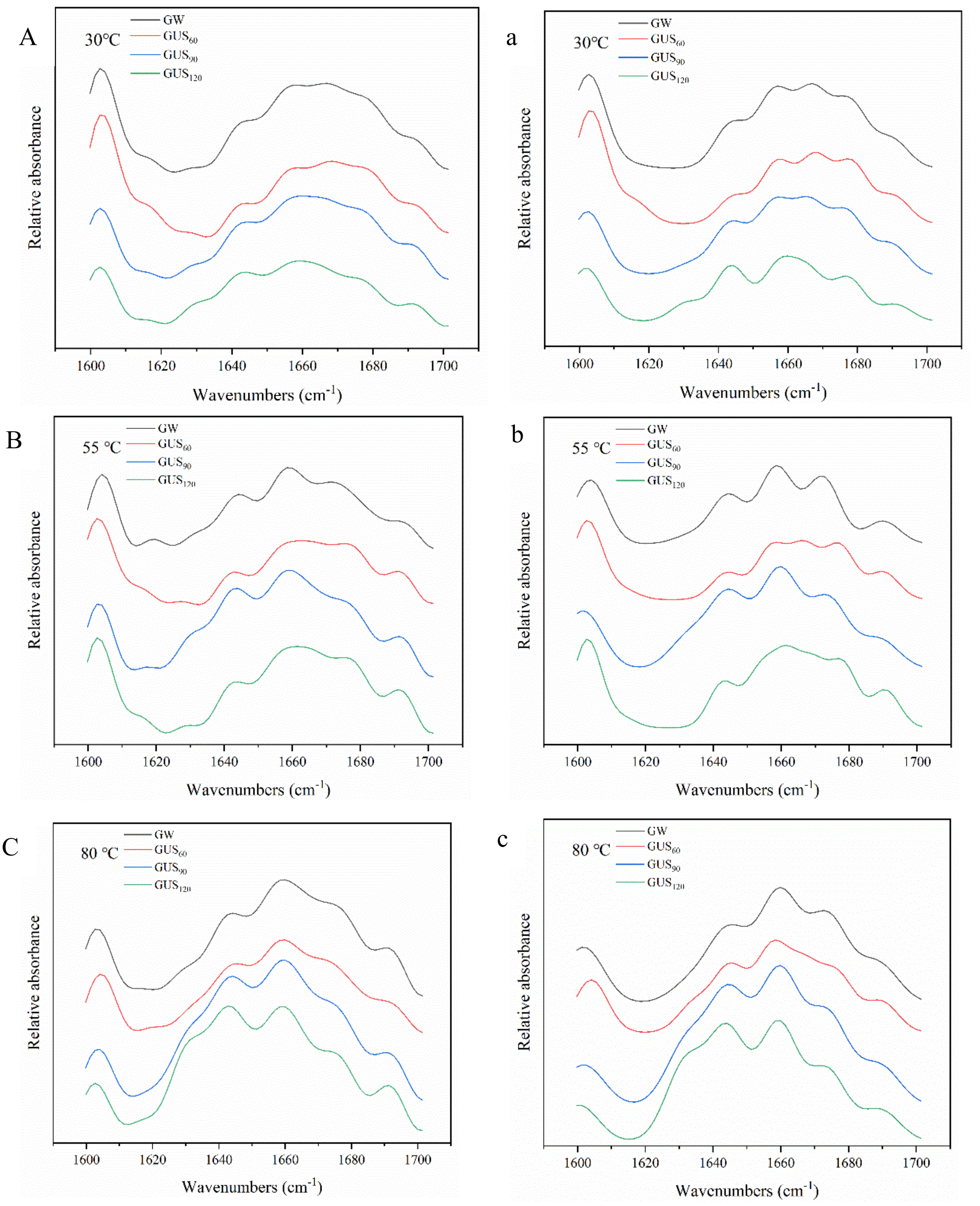

Figure S1. FTIR spectra in the amide I region (1600-1700 cm^{-1}) and deconvoluted component peaks of gluten samples. (A-C) FTIR spectra at 30, 55, and 80 °C; (a-c) deconvoluted FTIR spectra at 30, 55, and 80 °C.
